# Application of Pathomic Features for Differentiating Dysplastic Cells in Patients with Myelodysplastic Syndrome

**DOI:** 10.3390/bioengineering11121230

**Published:** 2024-12-05

**Authors:** Youngtaek Hong, Seri Jeong, Min-Jeong Park, Wonkeun Song, Nuri Lee

**Affiliations:** 1 CONNECT-AI Research Center, Yonsei University College of Medicine, Seoul 03764, Republic of Korea; hyt0205@gmail.com; 2 Department of Laboratory Medicine, Kangnam Sacred Heart Hospital, Hallym University College of Medicine, Seoul 07440, Republic of Korea; hehebox73@hallym.or.kr (S.J.); mjpark@hallym.or.kr (M.-J.P.); swonkeun@hallym.or.kr (W.S.)

**Keywords:** myelodysplastic syndromes, machine learning in pathology, bone marrow analysis, bone marrow neoplasms

## Abstract

Myelodysplastic syndromes (MDSs) are a group of hematologic neoplasms accompanied by dysplasia of bone marrow (BM) hematopoietic cells with cytopenia. Recently, digitalized pathology and pathomics using computerized feature analysis have been actively researched for classifying and predicting prognosis in various tumors of hematopoietic tissues. This study analyzed the pathomic features of hematopoietic cells in BM aspiration smears of patients with MDS according to each hematopoietic cell lineage and dysplasia. We included 24 patients with an MDS and 21 with normal BM. The 12,360 hematopoietic cells utilized were to be classified into seven types: normal erythrocytes, normal granulocytes, normal megakaryocytes, dysplastic erythrocytes, dysplastic granulocytes, dysplastic megakaryocytes, and others. Four hundred seventy-six pathomic features quantifying cell intensity, shape, and texture were extracted from each segmented cell. After comparing the combination of feature selection and machine learning classifier methods using 5-fold cross-validation area under the receiver operating characteristic curve (AUROC), the quadratic discriminant analysis (QDA) with gradient boosting decision tree (AUROC = 0.63) and QDA with eXtreme gradient boosting (XGB) (AUROC = 0.64) showed a high AUROC combination. Through a feature selection process, 30 characteristics were further analyzed. Dysplastic erythrocytes and granulocytes showed lower median values on heatmap analysis compared to that of normal erythrocytes and granulocytes. The data suggest that pathomic features could be applied to cell differentiation in hematologic malignancies. It could be used as a new biomarker with an auxiliary role for more accurate diagnosis. Further studies including prediction survival and prognosis with larger cohort of patients are needed.

## 1. Introduction

Myelodysplastic syndromes (MDSs) represent a collection of clonal bone marrow disorders characterized by ineffective hematopoiesis, resulting in morphological dysplasia of hematopoietic cells and peripheral blood cytopenias [1,2,3]. Although the importance of genomic features is growing in the diagnosis of MDSs, dysplastic morphology remains a crucial element in confirming the diagnosis [4]. However, the diagnostic and quantification processes for MDS dysplasia inherently involve subjective components, posing interpretative challenges. Furthermore, variations among observers can occur when assessing mild, borderline cases [1,5].

Recently, in the field of medical imaging analysis, research in radiomics, which enables the extraction of various quantitative and statistical characteristics of lesions through image analysis, has been actively conducted [6]. Pathomics utilizes radiomics parameters across various pathological tissues, enabling the derivation of objective indicators for the characteristics of diverse tissues [7]. Currently, pathomic features are actively researched in the field of oncology diagnosis, treatment efficacy assessment, and prognosis prediction in various tissues [8,9,10,11,12,13]. Nevertheless, research in the field of pathomics has not yet been sufficiently conducted due to the challenges associated with acquiring and analyzing bone marrow tissue. These difficulties include the invasive nature of bone marrow extraction procedures, which can limit sample availability; the heterogeneity of bone marrow samples, which complicates the standardization of data; and the need for high-resolution imaging to accurately capture cellular details. Additionally, the complex and variable morphology of bone marrow cells poses significant challenges for developing robust deep learning algorithms, requiring extensive annotation and validation to ensure accuracy and reliability in automated analyses [14,15]. Pathomics analysis in bone marrow samples remains an unexplored area, and it is expected to be utilized in understanding the characteristics of lesions, in pathophysiology, and in prognosis prediction in MDSs. As an emerging field that is starting to gain attention, it merges into the broader multi-omics framework, offering detailed insights into the structural alterations of tissues at a microscopic scale. Compiling pathomic data at the patient level will be crucial for effectively incorporating pathomics into clinical prognostic models [16].

We have previously developed an algorithm through earlier research that uses deep learning to detect hematopoietic cells in bone marrow aspirate slides and differentiate between dysplastic and normal cells in each cell lineage [17]. In the previous study, we classified the hematopoietic cells in bone marrow aspiration slide into eight categories (normal erythrocytes, normal granulocytes, normal megakaryocytes, dysplastic erythrocytes, dysplastic granulocytes, dysplastic megakaryocytes, blasts, and others) using the convolutional neural network (CNN) method with an accuracy of 0.912 to 0.993. We have demonstrated the potential of the developed algorithm as an auxiliary tool for diagnosing patients with MDS for shortening the time for diagnosis and standardizing visual reading.

This study aimed to identify hematopoietic cells in Korean patients with MDS and extract various pathomic signatures to analyze the characteristics of dysplastic and normal hematopoietic cells by lineage. Using a machine learning approach incorporating feature selection and cross-validated model evaluation, we examined the potential for distinguishing normal from dysplastic cells based on pathomic features of myelodysplastic cells. This approach provides insights into the potential for pathomic feature-based discrimination and informs directions for future research.

## 2. Materials and Methods

### 2.1. Dataset Preparation and Proposed Framework

**Figure 1:**
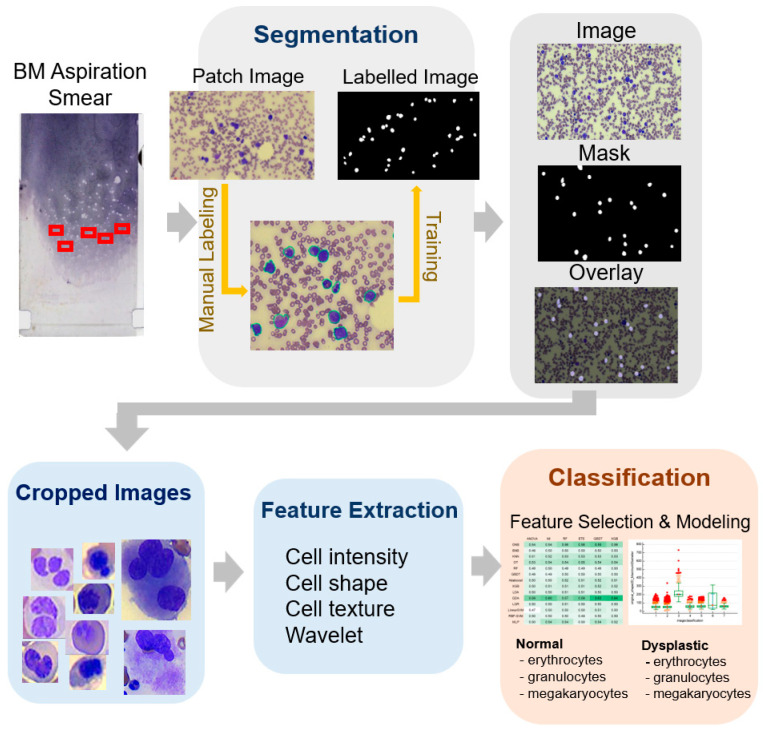
Workflow of the classification task of dysplastic cells in this study.

The workflow of this study is illustrated in Figure 1. A total of 24 patients diagnosed with an MDS and 21 normal bone marrow slides were included. The diagnosis of myelodysplastic syndrome was conducted according to the WHO 2016 MDS diagnostic criteria [18]. Normal bone marrow was designated from patients undergoing lymphoma routine staging work-ups, specifically those without any hematologic malignancy or reactive marrow findings and with no peripheral blood dilution. Wright–Giemsa staining was performed on bone marrow aspiration, and the comprehensive scanning of the bone marrow aspiration slide was conducted using Motic Digital Slide Assistant software version 1.0.7.61. (Motic China Group Co., Ltd., Xiamen, China). Automated identification and segmentation of nucleated cells in bone marrow aspiration slides were conducted following the previous study [17]. Briefly, the segmentation task was trained and tested with U-net, one of the convolutional network architectures, using patched bone marrow aspiration slide images. For the creation of patch images, areas with well-spread nucleated cells were manually selected and captured. A total of 11,000 patch images were produced. Manual labeling was conducted on 946 nucleated cells, and a segmentation algorithm was developed for cell detection within these patch images. The research adhered to the Declaration of Helsinki guidelines and received approval from the Institutional Review Board (IRB) of Kangnam Sacred Heart Hospital (IRB No. HKS 2021-07-023-008). Due to the study’s anonymized nature, the IRB waived the requirement for informed consent.

### 2.2. Pathomic Feature Extraction

The open-source Python package Pyradiomics was utilized to extract the pathomic features [19]. After image segmentation with an annotated mask, discretization and preprocessing of image of the segmented image was performed and applied to the first-, second-, or higher-order (texture) statistics. From bone marrow aspiration slides, 476 pathomic features were extracted from the segmented cell images of the identified nucleated cells. The radiomic features consisted of 9 shape features; 18 first-order statistical features; and 24 gray-level co-occurrence matrix (GLCM), 16 gray-level run-length matrix (GLRLM), 16 gray-level size zone matrix (GLSZM), 14 gray-level dependence matrix (GLDM), and 5 neighboring gray tone difference matrix (NGTDM) features. Four wavelet filters were applied to the image and extracted the same set of radiomic features from the wavelet response image. The GLCM was constructed using four directions: 0°, 45°, 90°, and 135°. The default offset of 1 was applied, and the GLCM was normalized by dividing each element by the total number of pixel pairs to ensure robust and reproducible feature extraction. The specific name of radiomic features are listed up in Supplementary Table S1. Using 1316 images each of image, Mask, and Overlay, we extracted pathomic features from a total of 12,360 segmented cells using 40X magnification patch images. The median number of patch images per patient was 26, while the median number of nucleated cells per patient, after segmentation and cropping, was 238.

### 2.3. Combination of Feature Selection and Modeling Methods

The extracted pathomic features were labeled for each cell according to lineage and the presence or absence of dysplasia in seven categories. The cell counts for each category are as follows: normal erythrocytes (NE), normal granulocytes (NG), normal megakaryocytes (NM), dysplastic erythrocytes (DE), dysplastic granulocytes (DG), and dysplastic megakaryocytes (DM), with 1533, 7902, 433, 716, 1217, and 540 cells, respectively. The labeling was performed by two hematologic pathologists with 30 and 11 years of experience, respectively. In cases of differing opinions, consensus labeling was conducted. To enhance label reliability, reviewers independently assigned labels, and any discrepancies were resolved through consensus discussions to reduce subjectivity and maintain consistency in the labeling process. To facilitate feature selection, (i) supervised feature selection was conducted to compare the extraction values for each cell class. Features were selected based on the statistical significance of the median value differences between normal and dysplastic cells across lineages and their adherence to a normal distribution. (ii) Combined validation was used to evaluate the potential utility of classification performance. Six well-known feature selection methods from existing literature were employed [20,21,22]. To differentiate between normal and dysplastic cells, 14 commonly used machine learning classifiers were applied. Each feature selection method was combined with all classifiers, yielding 84 cross-combinations. For each combination, 5-fold cross-validation results were calculated using the AUROC.

### 2.4. Statistical and Data Analysis

To assess the statistical significance of differences between the characteristics of MDS patients and the normal bone marrow group, the Mann–Whitney statistical method was utilized. Additionally, the statistical significance of differences between the extracted pathomic features across the seven cell classes, as well as the characteristics of normal versus dysplastic cells within each lineage, was evaluated using the Mann–Whitney test. Furthermore, a radar chart was created for the classification of dysplastic cells according to cell lineage, providing a visual representation of the characteristics for each lineage. All statistical analyses were conducted using Medcalc version 15.0 (MedCalc Software, Ostend, Belgium) and R version 4.1.0 (R Foundation for Statistical Computing).

## 3. Results

### 3.1. Demographics of Cohorts and Data Resource

In this study, a total of 45 patients were enrolled, including 24 with an MDS and 21 with normal bone marrow (Table 1). The median ages of the patients with MDSs and normal bone marrow were 70.5 (interquartile range (IQR) = 55.5–77.0) and 64.0 (IQR = 46.8–78.3), respectively. The number of male and female patients was 14 and 10 in the MDS group, and 12 and 9 in the normal bone marrow group, respectively. There was no statistical difference between the two groups in age and gender ratio. In patients with MDS, the median hemoglobin value was significantly lower at 8.5 g/dL compared to 13.3 g/dL in the normal group. The white blood cells (WBC) (3005 vs. 6560/µL) and platelet (94.5 vs. 232.0 × 10^3^/µL) counts were also significantly lower in the MDS group compared to that in the normal group. Lactate dehydrogenase (LD) levels were significantly higher in the MDS group compared to that in the normal group (260.0 vs. 183.0 IU/L). There was no difference in prothrombin time (PT) or activated partial thromboplastin time (aPTT), and other test results such as aspartate aminotransferase (AST), alanine aminotransferase (ALT), blood urea nitrogen (BUN), creatinine (Cr), and folate also showed no significant differences between the groups. Among patients with MDS, 33.3% each were classified under refractory anemia with excess blasts-2 (EB-2) and multilineage dysplasia (MLD), followed by 16.7% under refractory anemia with excess blasts-1 (EB-1), and 4.2% each under single lineage dysplasia (SLD), refractory multilineage dysplasia (R-MLD), myelodysplastic syndrome-unclassified (MDS-U), and therapy-related myelodysplastic syndrome (t-MDS).

### 3.2. Feature Extraction and Correlation for First Order

**Figure 2:**
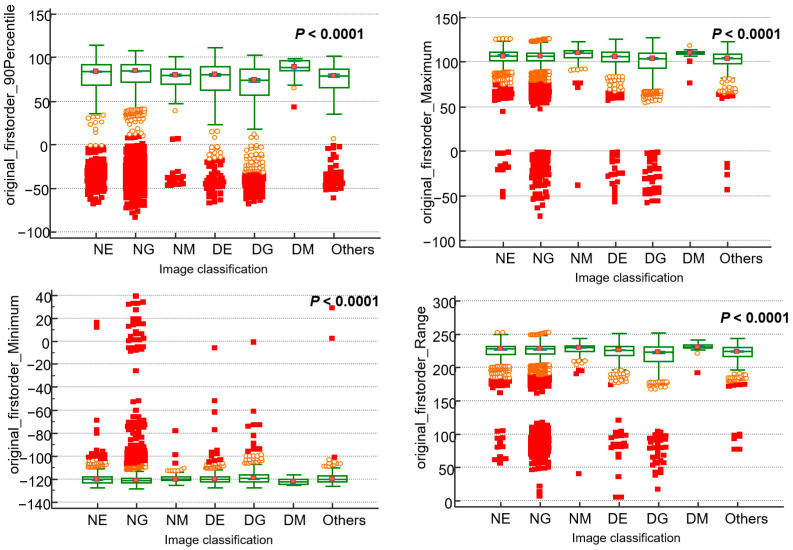
Comparison for pathomic features according to cell classification in patients with myelodysplastic syndrome. The x-axis categorizes cells into different types: dysplastic granulocyte (DG), others, dysplastic erythrocyte (DE), normal erythrocyte (NE), normal granulocyte (NG), dysplastic megakaryocyte (DM), normal megakaryocyte (NM), and others.

Extraction was performed for a total of 476 features, including 18 first-order statistical features and 24 GLCM, 16 GLRLM, 16 GLSZM, 14 GLDM, and 5 NGTDM features. Among these, the first-order statistical features and their median and interquartile values are described in Table 2. When comparing the values of normal and dysplastic cells by each cell’s lineage, the 90th percentile value of first-order statistics was significantly lower in dysplastic erythrocytes (DE, median 80.6, IQR = 62.9–89.8) and granulocytes (DG, median 74.0 IQR = 57.0–86.7) than in normal erythrocytes (NE, median 84.0, IQR = 68.5–91.7) and granulocytes (NG, 84.8, IQR = 71.6–92.0), respectively. Meanwhile, megakaryocytes showed significantly lower values in normal megakaryocytes (NM, median 80.0, IQR = 70.0–87.0) compared to that in dysplastic megakaryocytes (DM, median 84.0, IQR = 68.5–91.7). First-order energy and total energy also showed significantly lower values in normal erythrocytes and granulocytes compared to that in their dysplastic forms (Figure 2). Items such as wavelet high–high (HH) dependence nonuniformity showed higher values in both NM and DM, with NM values being higher than DM. When correlation analysis was performed for first-order features, variance and mean absolute deviation showed the highest correlation at 0.977, followed by robust mean absolute deviation (r = 0.939) and interquartile range (r = 0.864), which also exhibited high correlations with each other (Supplementary Figure S1).

### 3.3. Feature Selection and Modeling

**Figure 3:**
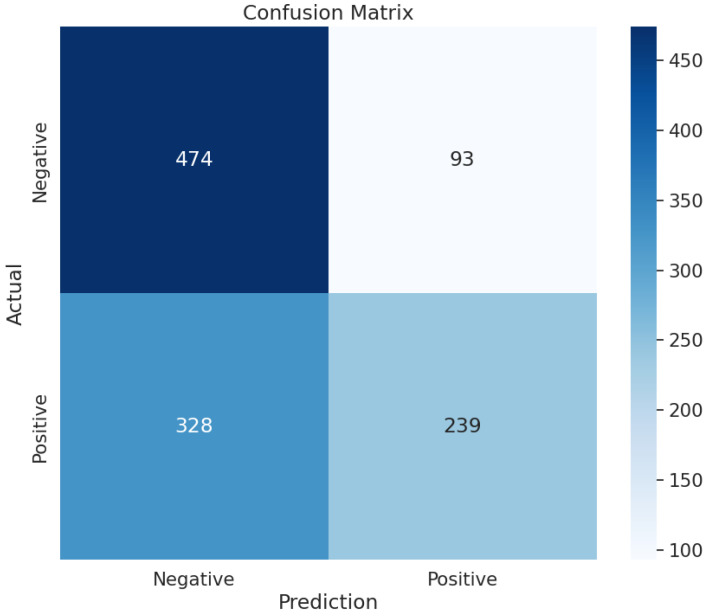
Confusion matrix illustrating the performance of the classification model. The matrix shows the number of true negatives (474), false positives (93), false negatives (328), and true positives (239). The rows represent the actual classes (negative and positive), while the columns represent the predicted classes. The color intensity reflects the magnitude of the values, with darker shades indicating higher counts.

We employed six feature selection methods: ANOVA, mutual information (MI), random forest (RF), ExtraTrees (ETE), gradient boosting decision tree (GBDT), and extreme gradient boost (XGB). Additionally, we conducted modeling using 14 different machine learning methods, resulting in the exploration of 64 combinations of feature selection and modeling methods. Exploring diverse combinations of feature selection techniques and machine learning models is a widely utilized approach in radiomics, particularly when working with handcrafted features [23]. Using feature selection methods, we calculated the information gain for all features and ranked them in descending order. Features were sequentially fed into the model, starting with those that had the highest information gain. Additional features were included until no further improvement in classification performance was observed. The results of comparing the combination of feature selection and ML classifier methods using 5-fold cross-validation area under the receiver operating characteristic curve (AUROC) are shown in Table 3. Eighty-four combinations of classification and feature selection methods were utilized in this study to find the combination with the highest performance. The combinations achieving a high AUROC included quadratic discriminant analysis (QDA) paired with GBDT, yielding an AUROC of 0.6315, an accuracy of 62.8%, a sensitivity of 42.2%, and a specificity of 83.6%. The corresponding confusion matrix (Figure 3) illustrates this performance, reflecting the model’s higher specificity compared to sensitivity. The top 30 features extracted from the QDA and GBDT combination are listed in Table 4.

### 3.4. Differences by Cell Lineage

**Figure 4:**
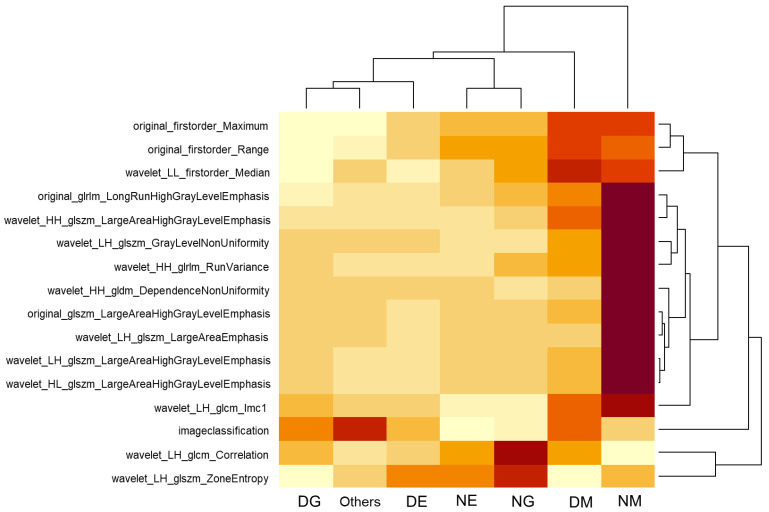
Heatmap of pathomic feature distribution in nucleated cells of patients with myelodysplastic syndromes. The x-axis denotes different cell types: DE, DG, and DM represent dysplastic erythrocytes, granulocytes, and megakaryocytes, respectively, while NE, NG, and NM represent normal erythrocytes, granulocytes, and megakaryocytes, respectively. Median values of pathomic features for each cell type are visualized, illustrating cell-specific patterns. Megakaryocytes (DM and NM) display higher median values compared to other lineages, likely reflecting intrinsic cellular characteristics. DE and DG show lower median values than their normal counterparts (NE and NG), with distinct patterns evident across dysplastic and normal cell types.

Additional analysis was conducted on the top 15 items among the top 30 features for each cell lineage’s normal and dysplastic cells. The median values of pathomic features for each cell type were visualized through a heatmap plot, and the results are shown in Figure 3. In the case of megakaryocytes, due to the cell’s intrinsic characteristics, they showed a higher median value compared to other cell lineages. Both erythrocytes and granulocytes exhibited differences between dysplastic and normal cells. Specifically, DE and DG showed lower median values compared to NE and NG, and distinct patterns were evident on the heatmap (Figure 4).

**Figure 5:**
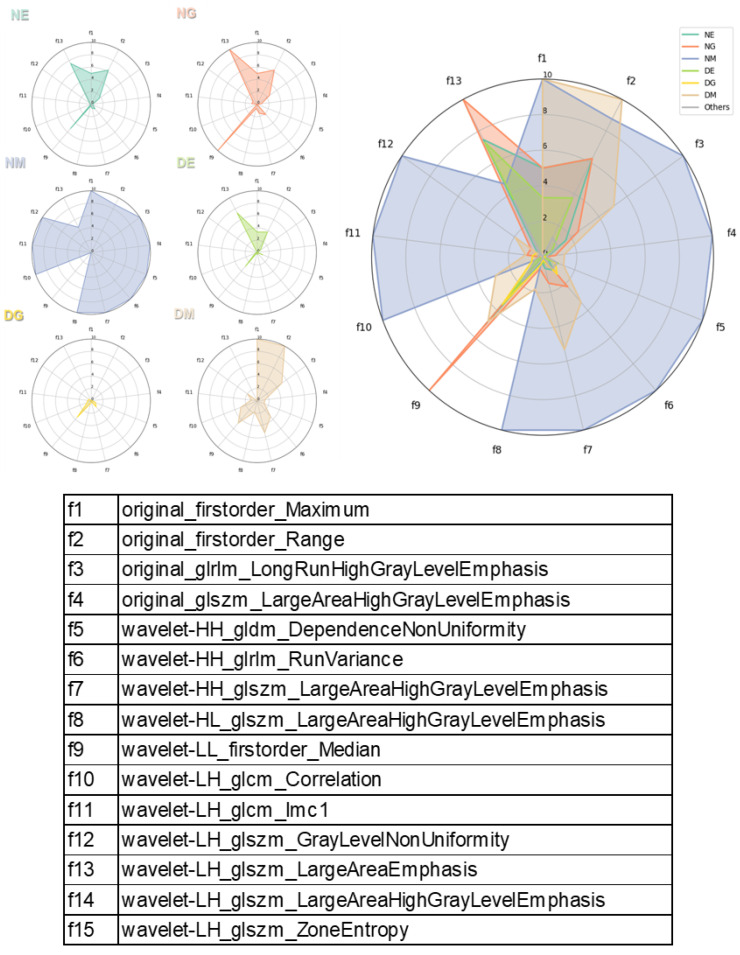
Radar chart for classification of dysplastic cells according to cell lineage.

Additionally, a radar plot was created to visually discriminate between the characteristics of each cell lineage based on their median values (Figure 5). For megakaryocytes, the normal form generally showed the highest values, except for f9 (wavelet-LL_firstorder_Median). In contrast, dysplastic forms had high values for f2 (original_firstorder_Range) and f1 (original_firstorder_Maximum) similar to normal, but lower values for f11 (wavelet-LH_glcm_Imc1) and f12 (wavelet-LH_glszm_GrayLevelNonUniformity), showing distinct characteristics. DG was notably the lowest in all items. DE also showed a lower area compared to NE, highlighting a distinguishable difference in dysplastic cells from normal. NE and NG displayed relatively similar radar plot patterns compared to other cell lineages. 

## 4. Discussion

In this study, various pathomic signature features of nucleated cells from patients with MDSs and normal BM aspiration smears were extracted and analyzed to explore their potential applications in MDS diagnosis and cell classification. The findings highlight the potential of pathomics for future cell classification.

In diagnosing hematologic malignancies, expert visual interpretation plays a pivotal role. While MDS diagnosis has traditionally relied on morphological evaluation, recent studies have examined the integration of advanced technologies as auxiliary tools [17,24,25,26,27,28,29,30]. Among these, the application of artificial intelligence in hematology research is steadily increasing, although studies specifically targeting MDS remain limited. Previous studies that applied deep learning for MDS cell classification reported varying levels of performance. For example, Lee et al. achieved sensitivity between 64.0% and 90.0% and specificity between 94.8% and 99.9% depending on the cell type [17], while Mori et al. demonstrated sensitivity and specificity of 91.0% and 97.7%, respectively, in distinguishing decreased granule scale [25]. However, to our knowledge, no previous studies have analyzed pathomics in bone marrow aspiration cell morphology and MDS diseases. This study is significant as the first to conduct a pathomic analysis specifically targeting patients with MDS. The pathomic features of each cell showed significant differences in median values across cell lineages and dysplastic status. Notably, normal erythrocytes and granulocytes versus dysplastic erythrocytes and granulocytes exhibited significant differences, with dysplastic cells showing lower values in many of the selected features. These distinctive differences were visually confirmed through heatmaps and radar plots.

Pathomic features, by quantifying and objectifying intrinsic cellular characteristics, hold potential for diverse applications. Recently, studies investigating the association between pathomic features and prognosis in various oncologic tissues have been actively conducted [8,9,13,31]. Radiomics has already become widely used and is one of the most extensively studied approaches for identifying imaging features linked to tumor pathophysiology [32,33,34,35,36]. The morphological characteristics of cells remain intrinsically important for diagnosis, even in an era where molecular assessments through massive sequencing are gaining prominence [37]. Image analysis, which enables classification based on underlying disease pathophysiology, underscores the clinical relevance of pathomic features, even within the context of molecular diagnostics. Pathomic features applied to tissue are anticipated to significantly impact both diagnosis and prognosis prediction in hematologic malignancies.

Quantified image analysis is also expected to contribute to the standardization and objectivity of diagnostic processes. Cell discrimination often relies on subjective visual assessment, which complicates standardization. Quantification is essential for achieving consistent interpretations unaffected by variability in expertise across specialists. Additionally, numerical values derived from various pathomic features facilitate objective interpretation, especially in areas that are visually challenging to differentiate. Visualization methods, such as the radar plots used in this study, can help clarify and illustrate the characteristics of individual cells. Previous studies have also attempted to quantify and analyze features like nucleated cells and fibrosis in bone marrow for diagnosing hematologic malignancies [38,39,40,41]. To our knowledge, the quantification of morphological characteristics extracted through pathomics of individual nucleated cells in bone marrow aspiration has not been attempted. However, dysplastic cells are expected to show distinctive differences that can be quantified as pathomic features, making this approach valuable.

A major challenge lies in selecting and utilizing various pathomic features for different diseases and establishing appropriate cutoff values. In radiomics, ongoing research focuses on evaluating feature selection methods and suitable classifiers for each specific disease [20,21]. Therefore, studies analyzing disease-specific pathomic feature characteristics are crucial for identifying pathomic features useful for each disease. Although artificial intelligence methods, such as unsupervised selection, have been recently applied, supervised selection that incorporates clinical experience remains valuable. This underscores the need for more studies comparing and evaluating different pathomic features, as done in this study. This research is anticipated to serve as a meaningful starting point for future investigations.

The limitations of this study include the small sample size, which precluded analysis of individual patient characteristics and application to patient-specific diagnosis. This limited sample size may constrain the generalizability and reliability of the conclusions, underscoring the importance of validation in larger, independent cohorts. Additionally, the lack of external data limits the scope of analysis and interpretation. Future research should aim to include a larger cohort of patients and apply pathomics at the patient level, rather than limiting the analysis to cell-level classification. Testing the model on an external dataset will also be essential to confirm its robustness and generalizability across different clinical settings. Follow-up research should incorporate data from multiple institutions to achieve standardization and address these limitations. Bone marrow aspirate samples, in particular, may vary in slide preparation and staining protocols across institutions. Therefore, comprehensive investigation and standardization of these methodologies are crucial to ensure reliable and reproducible diagnostic applications. In addition, this study employed PyRadiomics for texture feature extraction using wavelet-based analysis. While transform- and mathematical modeling-based techniques provide valuable insights [42], their use has been limited due to practical constraints, including the risk of overfitting with a small dataset. Future studies will address these limitations by expanding the dataset and incorporating broader transform-based approaches for a more comprehensive analysis of texture features. Finally, this study lacks systematic documentation of inter-reviewer labeling discrepancies. Although consensus discussions were used to resolve differences, the absence of quantitative tracking limits the ability to rigorously assess initial inter-rater variability. Future studies will implement structured discrepancy tracking to enhance label reliability assessment.

This study was restricted to cell-level classification, without expanding to patient-level diagnosis. Future research should progress from cellular-level discrimination towards comprehensive MDS diagnosis at the individual patient level. We intend to increase the sample size in subsequent studies to further validate these findings and enhance clinical applicability for patient-specific diagnostic frameworks. Future analyses that incorporate deep learning methods are anticipated to demonstrate improved performance, necessitating further research in this area. Similar approaches are being explored in radiology to enhance performance [43]. The combination of machine learning and deep learning has recently gained attention for classification tasks [8]. Applying this combination to optimize feature selection and classification is anticipated to yield improved results. Additionally, subsequent studies should integrate genomic data, expanding research into the field of pathogenomics.

## 5. Conclusions

In conclusion, this study demonstrates the clinical relevance of applying pathomic features to cell differentiation in hematologic malignancies, particularly in patients diagnosed with MDS. Pathomic features are expected to be actively investigated in nucleated cells of the bone marrow. As an objective and standardized tool, pathomics holds the potential to quantify bone marrow cell interpretation and diagnosis. It is anticipated that pathomics could be utilized as a novel biomarker, potentially playing an auxiliary role in enhancing diagnostic accuracy. While the performance obtained in this study is not yet sufficient for clinical application, we consider this work a foundational step toward future advancements. Moreover, this study holds particular significance as one of the first to explore pathomics in hematology. Building on this foundation, the research team plans to pursue follow-up studies aimed at achieving improved outcomes.

## Figures and Tables

**Table 1:** Baseline characteristics of cohorts.

	Classification		*p*-Value
	Myelodysplasia Syndromes (MDSs) (*n* = 24)	Normal Bone Marrow (*n* = 21)	
Age	70.5 (55.5–77.0)	64.0 (46.8–78.3)	0.3276
Sex (male:female)	14:10	12:9	0.9364
Aspartate aminotransferase (U/L)	26.0 (17.3–36.3)	20.0 (16.0–30.8)	0.2738
Alanine aminotrasnferase (U/L)	22.0 (11.0–42.8)	17.0 (12.8–22.3)	0.5331
Blood urea nitrogen (BUN, mg/dL)	13.5 (11.8–20.7)	13.5 (12.5–17.7)	0.8693
Creatinine (mg/dL)	0.75 (0.56–0.97)	0.82 (0.69–0.91)	0.8416
Folate (ng/mL)	6.6 (5.1–10.2)	14.5 (9.0–21.7)	0.0091
Lactate dehydrogenase (IU/L)	260.0 (216.0–337.0)	183.0 (153.8–197.5)	<0.0001
Hemoglobin (g/dL)	8.5 (7.9–9.1)	13.3 (11.9–14.1)	<0.0001
White blood cells (/uL)	3005 (1970–3780)	6560 (5515–8288)	<0.0001
Platelet (10^3^/uL)	94.5 (45.0–157.5)	232.0 (183.0–296.8)	0.0001
Prothrombin time (s)	12.5 (11.6–13.7)	12.4 (11.5–13.2)	0.5009
aPTT (s)	30.4 (28.4–33.8)	32.0 (28.3–34.8)	0.6398
MDS classification Single lineage dysplasia Multi-lineage dysplasia (MLD) Excess blast-1 Excess blast-2 Ring sideroblasts MLD Unclassifiable Therapy-related MLD	1 (4.2%) 8 (33.3%) 4 (16.7%) 8 (33.3%) 1 (4.2%) 1 (4.2%) 1 (4.2%)	N/A	N/A

**Table 2:** First-order statistical features of normal and dysplastic cells in erythrocytes, granulocytes, and megakaryocytes.

	Image Classification								
First Order	Erythrocytes			Granulocytes			Megakaryocytes		
	Normal	Dysplastic	*p*	Normal	Dysplastic	*p*	Normal	Dysplastic	*p*
10 Percentile	−101 (−107.6 to −91.8	−102 (−107.2 to −93.0)	0.6494	−102 (−108.1 to −91.3)	−100.4 (−106.6 to −92.2)	0.0035	−87.3 (−98.0 to −71.5)	−94.5 (−108.0 to −84.5)	0.0672
90 Percentile	84 (68.5 to 91.7)	80.6 (62.9 to 89.8)	0.0001	84.8 (71.6 to 92.0)	74 (57.0 to 86.7)	<0.0001	80.0 (70.0 to 87.0)	89.2 (85.2 to 96.8)	0.0004
Energy	808,744 (635,607 to 1,037,648)	884,340 (691,798 to 1,118,195)	<0.0001	813,222 (648,916 to 997,607)	884,137 (687,053 to 1,120,851)	<0.0001	2,427,583 (1,838,880 to 3,171,995)	931,679 (530,292 to 3,294,565)	0.007
Entropy	2.7 (2.4 to 2.9)	2.7 (2.5 to 2.9	0.389	2.7 (2.5 to 2.8)	2.6 (2.4 to 2.8)	0.0001	2.7 (2.5 to 2.8)	2.8 (2.6 to 2.8)	0.3043
Interquartile Range	123 (56.9 to 147.0)	122 (57.9 to 144.8)	0.4963	123 (53.3 to 147.5)	115 (43 to 141)	<0.0001	102.8 (46.8 to 129.0)	81 (54.8 to 129.3)	0.9192
Kurtosis	1.9 (1.5 to 2.8)	1.8 (1.5 to 2.7	0.488	1.9 (1.5 to 2.8)	1.9 (1.5 to 3.2)	0.0002	2.1 (1.5 to 3.1)	2.2 (1.8 to 4.0)	0.3259
Maximum	107 (102 to 111)	106 (100 to 111)	0.1449	107 (102 to 110)	104 (93 to 110)	<0.0001	110 (105 to 113)	110 (109 to 112)	0.8707
Mean	−6.8 (−30 to 14.3)	−12.7 (−35.2 to 8.2)	0.0001	−4.7 (−27.6 to 16.2)	−20.4 (−43.5 to 2.3)	<0.0001	8.1 (−13.9 to 22.8	12.0 (−3.9 to 30.3	0.2255
Mean Absolute Deviation	60.6 (47.5 to 70.3)	60.5 (47.4 to 69.4)	0.4078	60.9 (48.0 to 70.6)	56.8 (41.8 to 67.5)	<0.0001	53.9 (42.7 to 63.8	52.9 (42.5 to 64.1)	0.8023
Median	−6 (−56 to 46)	−33 (−60 to 41)	0.0002	7.8 (−54 to 49)	−45 (−62 to 35)	<0.0001	31.5 (−40 to 44)	40.0 (−29.4 to 49.5)	0.527
Minimum	−120 (−123 to −118)	−120 (−122 to −118)	0.0036	−121 (−123 to −119)	−119 (−122 to −116)	<0.0001	−120 (−121 to −118)	−122 (−124 to −120)	0.0015
Range	228 (220 to 232)	226 (218 to 232)	0.0353	228 (221 to 232)	223 (210 to 231)	<0.0001	230 (224 to 233)	231 (229.3 to 234.5)	0.1164
Robust Mean Absolute Deviation	48.8 (32.4 to 60.7)	48.4 (32.6 to 60.1)	0.6169	49.1 (32.7 to 61.2)	44.8 (26.3 to 58.0)	<0.0001	41.5 (28.3 to 54.0)	38.0 (25.9 to 53.9)	0.9235
Root Mean Squared	74.7 (68.3 to 80.6)	74.9 (68.4 to 80.3)	0.6922	75.1 (68.6 to 81.1)	73.6 (67.4 to 78.8)	<0.0001	67.1 (60.1 to 73.7)	71.1 (64.2 to 76.5)	0.0663
Skewness	−0.02 (−0.6 to 0.5)	0.07 (−0.41 to 0.64)	0.0021	−0.09 (−0.65 to 0.49)	0.25 (−0.35 to 0.86)	<0.0001	−0.38 (−0.86 to 0.12)	−0.6 (−1.1 to −0.01)	0.1881
Total Energy	808,744 (635,607 to 1,037,648)	884,339 (691,798 to 1,118,195)	<0.0001	813,222 (648,916 to 997,607)	884,137 (687,053 to 1,120,851)	<0.0001	2,427,583 (1,838,880 to 3,171,995)	931,679 (530,292 to 3,294,565)	0.007
Uniformity	0.18 (0.16 to 0.22)	0.18 (0.15 to 0.21	0.3958	0.18 (0.16 to 0.22)	0.19 (0.16 to 0.23)	0.0001	0.19 (0.16 to 0.23)	0.17 (0.16 to 0.19)	0.1027
Variance	4770 (3539 to 5805)	4691 (3459 to 5641)	0.2284	4807 (3603 to 5896)	4305 (2962 to 5413)	<0.0001	3911 (2946 to 4916)	4369 (3274 to 5187)	0.2446

**Table 3:** Combination of feature selection and modeling methods by 5-fold cross validation results with area under receiver operating characteristics curve.

	ANOVA	MI	RF	ETE	GBDT	XGB
GNB	0.54	0.54	0.56	0.58	0.59	0.56
BNB	0.49	0.50	0.50	0.50	0.50	0.50
KNN	0.51	0.52	0.53	0.53	0.53	0.53
DT	0.53	0.54	0.54	0.55	0.54	0.54
RF	0.49	0.50	0.49	0.49	0.49	0.50
GBDT	0.49	0.49	0.50	0.50	0.50	0.50
Adaboost	0.50	0.50	0.52	0.51	0.52	0.51
XGB	0.50	0.51	0.51	0.51	0.52	0.52
LDA	0.50	0.50	0.51	0.51	0.50	0.50
QDA	0.59	0.60	0.57	0.59	0.63	0.64
LGR	0.50	0.50	0.51	0.50	0.50	0.50
Linear-SVM	0.47	0.50	0.50	0.50	0.51	0.50
RBF-SVM	0.50	0.50	0.50	0.49	0.50	0.50
MLP	0.50	0.54	0.54	0.50	0.54	0.52

Abbreviations: ANOVA: analysis of variance; MI: mutual information; RF: random forest; ETE: ExtraTrees; GBDT: gradient boosting decision tree; XGB: extreme gradient Boost; GNB: Gaussian naïve Bayes; BNB: Bernoulli naïve Bayes; LDA: linear discriminant analysis; QDA: quadratic discriminant analysis; LGR: logistic regression; MLP: multi-layer perceptron classifier.

**Table 4:** Top 30 features on the combination of gradient boosting decision tree (GBDT) and quadratic discriminant analysis (QDA).

wavelet-HH_gldm_DependenceNonUniformity original_firstorder_Maximum original_glszm_LargeAreaHighGrayLevelEmphasis wavelet-LH_glszm_ZoneEntropy wavelet-LH_glszm_LargeAreaLowGrayLevelEmphasis original_firstorder_Range wavelet-LH_glcm_Imc1 wavelet-HH_glrlm_RunVariance wavelet-LH_glszm_GrayLevelNonUniformity wavelet-HL_glszm_LargeAreaLowGrayLevelEmphasis wavelet-HH_glszm_LargeAreaLowGrayLevelEmphasi wavelet-LH_glszm_LargeAreaEmphasis original_glrlm_LongRunHighGrayLevelEmphasis wavelet-LL_firstorder_Median wavelet-LH_glcm_Correlation original_firstorder_90Percentile original_shape2D_MinorAxisLength wavelet-LH_glrlm_RunVariance wavelet-HL_glrlm_RunVariance wavelet-LH_glszm_LargeAreaHighGrayLevelEmphasis wavelet-HL_glszm_GrayLevelNonUniformity original_firstorder_Minimum wavelet-LL_ngtdm_Contrast wavelet-LH_ngtdm_Strength wavelet-HH_firstorder_Maximum original_glcm_Imc2 wavelet-LH_glszm_ZoneVariance wavelet-HL_glcm_Imc1 wavelet-HL_glcm_InverseVariance wavelet-HL_gldm_DependenceNonUniformity

## Data Availability

All relevant database described in this study has been deposited on the Harvard Dataverse website (Lee, Nuri, 2024, “Bone marrow aspiration pathomics in myelodysplastic syndrome”, https://doi.org/10.7910/DVN/ELW1CV, Harvard Dataverse).

## Supplementary Materials

The following are available online at https://www.mdpi.com/article/10.3390/bioengineering11121230/s1, Figure S1: Pearson correlation coefficient of first-order features extracted from hematopoietic cells in bone marrow aspiration in patients with myelodysplastic syndrome; Table S1: Comprehensive List of Pathomic Features.
