# Supplementary material for: Application of Pathomic Features for Differentiating Dysplastic Cells in Patients with Myelodysplastic Syndrome
Source: Bioengineering (Basel). 2024 Dec 5;11(12):1230. doi: 10.3390/bioengineering11121230 (PMC11673167; doi:10.3390/bioengineering11121230)
Supplement: Supplementary file 1 [file bioengineering-11-01230-s001.zip › bioengineering-3316327-supplementary.pdf]

Supplementary information

Supplementary Figure S1. Pearson correlation coefficient of first-order features extracted from hematopoietic cells in bone marrow aspiration in patients with myelodysplastic syndrome

Supplementary Table S1. Comprehensive List of Pathomic Features

Supplementary Figure S1. Pearson correlation coefficient of first-order features extracted from hematopoietic cells in bone marrow aspiration in patients with myelodysplastic syndrome

1.0 -1.0

	original_firstorder_Variance	original_firstorder_MeanAbsoluteDeviation	original_firstorder_RobustMeanAbsoluteDeviation	original_firstorder_InterquartileRange	original_firstorder_90Percentile	original_firstorder_Maximum	original_firstorder_Range	original_firstorder_RootMeanSquared	original_firstorder_Energy	original_firstorder_TotalEnergy	original_firstorder_Mean	original_firstorder_Median	original_firstorder_Entropy	original_firstorder_Minimum	original_firstorder_10Percentile	original_firstorder_Kurtosis	original_firstorder_Skewness	original_firstorder_Uniformity
original_firstorder_Variance		0.977	0.939	0.864	0.679	0.484	0.546	0.704	0.213	0.213	0.186	0.159	0.431	-0.375	-0.547	-0.643	-0.141	-0.493
original_firstorder_MeanAbsoluteDeviation	0.977		0.981	0.915	0.674	0.471	0.527	0.603	0.202	0.202	0.174	0.143	0.529	-0.356	-0.536	-0.714	-0.129	-0.584
original_firstorder_RobustMeanAbsoluteDeviation	0.939	0.981		0.946	0.588	0.378	0.425	0.554	0.195	0.195	0.124	0.104	0.503	-0.290	-0.505	-0.688	-0.091	-0.561
original_firstorder_InterquartileRange	0.864	0.915	0.946		0.521	0.334	0.376	0.484	0.174	0.174	0.100	0.083	0.517	-0.257	-0.452	-0.643	-0.073	-0.566
original_firstorder_90Percentile	0.679	0.674	0.588	0.521		0.642	0.596	0.229	0.057	0.057	0.635	0.466	0.533	-0.184	0.010	-0.568	-0.564	-0.540
original_firstorder_Maximum	0.484	0.471	0.378	0.334	0.642		0.919	0.220	0.123	0.123	0.377	0.260	0.515	-0.266	-0.048	-0.196	-0.169	-0.514
original_firstorder_Range	0.546	0.527	0.425	0.376	0.596	0.919		0.311	0.161	0.161	0.284	0.205	0.542	-0.624	-0.205	-0.211	-0.146	-0.549
original_firstorder_RootMeanSquared	0.704	0.603	0.554	0.484	0.229	0.220	0.311		0.283	0.283	-0.199	-0.163	-0.058	-0.324	-0.568	-0.177	0.208	-0.048
original_firstorder_Energy	0.213	0.202	0.195	0.174	0.057	0.123	0.161	0.283		1.000	-0.092	-0.073	0.021	-0.151	-0.202	-0.084	0.104	-0.036
original_firstorder_TotalEnergy	0.213	0.202	0.195	0.174	0.057	0.123	0.161	0.283	1.000		-0.092	-0.073	0.021	-0.151	-0.202	-0.084	0.104	-0.036
original_firstorder_Mean	0.186	0.174	0.124	0.100	0.635	0.377	0.284	-0.199	-0.092	-0.092		0.900	0.166	0.051	0.472	-0.114	-0.929	-0.129
original_firstorder_Median	0.159	0.143	0.104	0.083	0.466	0.260	0.205	-0.163	-0.073	-0.073	0.900		0.100	0.014	0.329	-0.078	-0.831	-0.064
original_firstorder_Entropy	0.431	0.529	0.503	0.517	0.533	0.515	0.542	-0.058	0.021	0.021	0.166	0.100		-0.305	-0.283	-0.611	-0.102	-0.960
original_firstorder_Minimum	-0.375	-0.356	-0.290	-0.257	-0.184	-0.266	-0.624	-0.324	-0.151	-0.151	0.051	0.014	-0.305		0.407	0.128	0.023	0.325
original_firstorder_10Percentile	-0.547	-0.536	-0.505	-0.452	0.010	-0.048	-0.205	-0.568	-0.202	-0.202	0.472	0.329	-0.283	0.407		0.455	-0.464	0.330
original_firstorder_Kurtosis	-0.643	-0.714	-0.688	-0.643	-0.568	-0.196	-0.211	-0.177	-0.084	-0.084	-0.114	-0.078	-0.611	0.128	0.455		0.137	0.628
original_firstorder_Skewness	-0.141	-0.129	-0.091	-0.073	-0.564	-0.169	-0.146	0.208	0.104	0.104	-0.929	-0.831	-0.102	0.023	-0.464	0.137		0.073
original_firstorder_Uniformity	-0.493	-0.584	-0.561	-0.566	-0.540	-0.514	-0.549	-0.048	-0.036	-0.036	-0.129	-0.064	-0.960	0.325	0.330	0.628	0.073	

Pearson correlation coefficient

Supplementary Table S1. Comprehensive List of Pathomic Features.

**Extracted Pathomic Features**
original_shape2D_MeshSurface
original_shape2D_PixelSurface
original_shape2D_Perimeter
original_shape2D_PerimeterSurfaceRatio
original_shape2D_Sphericity
original_shape2D_SphericalDisproportion
original_shape2D_MaximumDiameter
original_shape2D_MajorAxisLength
original_shape2D_MinorAxisLength
original_shape2D_Elongation
original_firstorder_10Percentile
original_firstorder_90Percentile
original_firstorder_Energy
original_firstorder_Entropy
original_firstorder_InterquartileRange
original_firstorder_Kurtosis
original_firstorder_Maximum
original_firstorder_MeanAbsoluteDeviation
original_firstorder_Mean
original_firstorder_Median
original_firstorder_Minimum
original_firstorder_Range
original_firstorder_RobustMeanAbsoluteDeviation
original_firstorder_RootMeanSquared
original_firstorder_Skewness
original_firstorder_TotalEnergy
original_firstorder_Uniformity
original_firstorder_Variance
original_glcm_Autocorrelation
original_glcm_JointAverage
original_glcm_ClusterProminence
original_glcm_ClusterShade
original_glcm_ClusterTendency
original_glcm_Contrast
original_glcm_Correlation
original_glcm_DifferenceAverage
original_glcm_DifferenceEntropy

original_glcm_DifferenceVariance
original_glcm_JointEnergy
original_glcm_JointEntropy
original_glcm_Imc1
original_glcm_Imc2
original_glcm_Idm
original_glcm_Idmn
original_glcm_Id
original_glcm_Idn
original_glcm_InverseVariance
original_glcm_MaximumProbability
original_glcm_SumEntropy
original_glcm_SumSquares
original_gldm_DependenceEntropy
original_gldm_DependenceNonUniformity
original_gldm_DependenceNonUniformityNormalized
original_gldm_DependenceVariance
original_gldm_GrayLevelNonUniformity
original_gldm_GrayLevelVariance
original_gldm_HighGrayLevelEmphasis
original_gldm_LargeDependenceEmphasis
original_gldm_LargeDependenceHighGrayLevelEmphasis
original_gldm_LargeDependenceLowGrayLevelEmphasis
original_gldm_LowGrayLevelEmphasis
original_gldm_SmallDependenceEmphasis
original_gldm_SmallDependenceHighGrayLevelEmphasis
original_gldm_SmallDependenceLowGrayLevelEmphasis
original_glrlm_GrayLevelNonUniformity
original_glrlm_GrayLevelNonUniformityNormalized
original_glrlm_GrayLevelVariance
original_glrlm_HighGrayLevelRunEmphasis
original_glrlm_LongRunEmphasis
original_glrlm_LongRunHighGrayLevelEmphasis
original_glrlm_LongRunLowGrayLevelEmphasis
original_glrlm_LowGrayLevelRunEmphasis
original_glrlm_RunEntropy
original_glrlm_RunLengthNonUniformity
original_glrlm_RunLengthNonUniformityNormalized
original_glrlm_RunPercentage
original_glrlm_RunVariance

original_glrlm_ShortRunEmphasis
original_glrlm_ShortRunHighGrayLevelEmphasis
original_glrlm_ShortRunLowGrayLevelEmphasis
original_glszm_GrayLevelNonUniformity
original_glszm_GrayLevelNonUniformityNormalized
original_glszm_GrayLevelVariance
original_glszm_HighGrayLevelZoneEmphasis
original_glszm_LargeAreaEmphasis
original_glszm_LargeAreaHighGrayLevelEmphasis
original_glszm_LargeAreaLowGrayLevelEmphasis
original_glszm_LowGrayLevelZoneEmphasis
original_glszm_SizeZoneNonUniformity
original_glszm_SizeZoneNonUniformityNormalized
original_glszm_SmallAreaEmphasis
original_glszm_SmallAreaHighGrayLevelEmphasis
original_glszm_SmallAreaLowGrayLevelEmphasis
original_glszm_ZoneEntropy
original_glszm_ZonePercentage
original_glszm_ZoneVariance
original_ngtdm_Busyness
original_ngtdm_Coarseness
original_ngtdm_Complexity
original_ngtdm_Contrast
original_ngtdm_Strength
wavelet-LH_firstorder_10Percentile
wavelet-LH_firstorder_90Percentile
wavelet-LH_firstorder_Energy
wavelet-LH_firstorder_Entropy
wavelet-LH_firstorder_InterquartileRange
wavelet-LH_firstorder_Kurtosis
wavelet-LH_firstorder_Maximum
wavelet-LH_firstorder_MeanAbsoluteDeviation
wavelet-LH_firstorder_Mean
wavelet-LH_firstorder_Median
wavelet-LH_firstorder_Minimum
wavelet-LH_firstorder_Range
wavelet-LH_firstorder_RobustMeanAbsoluteDeviation
wavelet-LH_firstorder_RootMeanSquared
wavelet-LH_firstorder_Skewness
wavelet-LH_firstorder_TotalEnergy

wavelet-LH_firstorder_Uniformity
wavelet-LH_firstorder_Variance
wavelet-LH_glcm_Autocorrelation
wavelet-LH_glcm_JointAverage
wavelet-LH_glcm_ClusterProminence
wavelet-LH_glcm_ClusterShade
wavelet-LH_glcm_ClusterTendency
wavelet-LH_glcm_Contrast
wavelet-LH_glcm_Correlation
wavelet-LH_glcm_DifferenceAverage
wavelet-LH_glcm_DifferenceEntropy
wavelet-LH_glcm_DifferenceVariance
wavelet-LH_glcm_JointEnergy
wavelet-LH_glcm_JointEntropy
wavelet-LH_glcm_Imc1
wavelet-LH_glcm_Imc2
wavelet-LH_glcm_Idm
wavelet-LH_glcm_Idmn
wavelet-LH_glcm_Id
wavelet-LH_glcm_Idn
wavelet-LH_glcm_InverseVariance
wavelet-LH_glcm_MaximumProbability
wavelet-LH_glcm_SumEntropy
wavelet-LH_glcm_SumSquares
wavelet-LH_gldm_DependenceEntropy
wavelet-LH_gldm_DependenceNonUniformity
wavelet-LH_gldm_DependenceNonUniformityNormalized
wavelet-LH_gldm_DependenceVariance
wavelet-LH_gldm_GrayLevelNonUniformity
wavelet-LH_gldm_GrayLevelVariance
wavelet-LH_gldm_HighGrayLevelEmphasis
wavelet-LH_gldm_LargeDependenceEmphasis
wavelet-LH_gldm_LargeDependenceHighGrayLevelEmphasis
wavelet-LH_gldm_LargeDependenceLowGrayLevelEmphasis
wavelet-LH_gldm_LowGrayLevelEmphasis
wavelet-LH_gldm_SmallDependenceEmphasis
wavelet-LH_gldm_SmallDependenceHighGrayLevelEmphasis
wavelet-LH_gldm_SmallDependenceLowGrayLevelEmphasis
wavelet-LH_glrlm_GrayLevelNonUniformity
wavelet-LH_glrlm_GrayLevelNonUniformityNormalized

wavelet-LH_glrlm_GrayLevelVariance
wavelet-LH_glrlm_HighGrayLevelRunEmphasis
wavelet-LH_glrlm_LongRunEmphasis
wavelet-LH_glrlm_LongRunHighGrayLevelEmphasis
wavelet-LH_glrlm_LongRunLowGrayLevelEmphasis
wavelet-LH_glrlm_LowGrayLevelRunEmphasis
wavelet-LH_glrlm_RunEntropy
wavelet-LH_glrlm_RunLengthNonUniformity
wavelet-LH_glrlm_RunLengthNonUniformityNormalized
wavelet-LH_glrlm_RunPercentage
wavelet-LH_glrlm_RunVariance
wavelet-LH_glrlm_ShortRunEmphasis
wavelet-LH_glrlm_ShortRunHighGrayLevelEmphasis
wavelet-LH_glrlm_ShortRunLowGrayLevelEmphasis
wavelet-LH_glszm_GrayLevelNonUniformity
wavelet-LH_glszm_GrayLevelNonUniformityNormalized
wavelet-LH_glszm_GrayLevelVariance
wavelet-LH_glszm_HighGrayLevelZoneEmphasis
wavelet-LH_glszm_LargeAreaEmphasis
wavelet-LH_glszm_LargeAreaHighGrayLevelEmphasis
wavelet-LH_glszm_LargeAreaLowGrayLevelEmphasis
wavelet-LH_glszm_LowGrayLevelZoneEmphasis
wavelet-LH_glszm_SizeZoneNonUniformity
wavelet-LH_glszm_SizeZoneNonUniformityNormalized
wavelet-LH_glszm_SmallAreaEmphasis
wavelet-LH_glszm_SmallAreaHighGrayLevelEmphasis
wavelet-LH_glszm_SmallAreaLowGrayLevelEmphasis
wavelet-LH_glszm_ZoneEntropy
wavelet-LH_glszm_ZonePercentage
wavelet-LH_glszm_ZoneVariance
wavelet-LH_ngtdm_Busyness
wavelet-LH_ngtdm_Coarseness
wavelet-LH_ngtdm_Complexity
wavelet-LH_ngtdm_Contrast
wavelet-LH_ngtdm_Strength
wavelet-HL_firstorder_10Percentile
wavelet-HL_firstorder_90Percentile
wavelet-HL_firstorder_Energy
wavelet-HL_firstorder_Entropy
wavelet-HL_firstorder_InterquartileRange

wavelet-HL_firstorder_Kurtosis
wavelet-HL_firstorder_Maximum
wavelet-HL_firstorder_MeanAbsoluteDeviation
wavelet-HL_firstorder_Mean
wavelet-HL_firstorder_Median
wavelet-HL_firstorder_Minimum
wavelet-HL_firstorder_Range
wavelet-HL_firstorder_RobustMeanAbsoluteDeviation
wavelet-HL_firstorder_RootMeanSquared
wavelet-HL_firstorder_Skewness
wavelet-HL_firstorder_TotalEnergy
wavelet-HL_firstorder_Uniformity
wavelet-HL_firstorder_Variance
wavelet-HL_glcm_Autocorrelation
wavelet-HL_glcm_JointAverage
wavelet-HL_glcm_ClusterProminence
wavelet-HL_glcm_ClusterShade
wavelet-HL_glcm_ClusterTendency
wavelet-HL_glcm_Contrast
wavelet-HL_glcm_Correlation
wavelet-HL_glcm_DifferenceAverage
wavelet-HL_glcm_DifferenceEntropy
wavelet-HL_glcm_DifferenceVariance
wavelet-HL_glcm_JointEnergy
wavelet-HL_glcm_JointEntropy
wavelet-HL_glcm_Imc1
wavelet-HL_glcm_Imc2
wavelet-HL_glcm_Idm
wavelet-HL_glcm_Idmn
wavelet-HL_glcm_Id
wavelet-HL_glcm_Idn
wavelet-HL_glcm_InverseVariance
wavelet-HL_glcm_MaximumProbability
wavelet-HL_glcm_SumEntropy
wavelet-HL_glcm_SumSquares
wavelet-HL_gldm_DependenceEntropy
wavelet-HL_gldm_DependenceNonUniformity
wavelet-HL_gldm_DependenceNonUniformityNormalized
wavelet-HL_gldm_DependenceVariance
wavelet-HL_gldm_GrayLevelNonUniformity

wavelet-HL_gldm_GrayLevelVariance
wavelet-HL_gldm_HighGrayLevelEmphasis
wavelet-HL_gldm_LargeDependenceEmphasis
wavelet-HL_gldm_LargeDependenceHighGrayLevelEmphasis
wavelet-HL_gldm_LargeDependenceLowGrayLevelEmphasis
wavelet-HL_gldm_LowGrayLevelEmphasis
wavelet-HL_gldm_SmallDependenceEmphasis
wavelet-HL_gldm_SmallDependenceHighGrayLevelEmphasis
wavelet-HL_gldm_SmallDependenceLowGrayLevelEmphasis
wavelet-HL_glrlm_GrayLevelNonUniformity
wavelet-HL_glrlm_GrayLevelNonUniformityNormalized
wavelet-HL_glrlm_GrayLevelVariance
wavelet-HL_glrlm_HighGrayLevelRunEmphasis
wavelet-HL_glrlm_LongRunEmphasis
wavelet-HL_glrlm_LongRunHighGrayLevelEmphasis
wavelet-HL_glrlm_LongRunLowGrayLevelEmphasis
wavelet-HL_glrlm_LowGrayLevelRunEmphasis
wavelet-HL_glrlm_RunEntropy
wavelet-HL_glrlm_RunLengthNonUniformity
wavelet-HL_glrlm_RunLengthNonUniformityNormalized
wavelet-HL_glrlm_RunPercentage
wavelet-HL_glrlm_RunVariance
wavelet-HL_glrlm_ShortRunEmphasis
wavelet-HL_glrlm_ShortRunHighGrayLevelEmphasis
wavelet-HL_glrlm_ShortRunLowGrayLevelEmphasis
wavelet-HL_glszm_GrayLevelNonUniformity
wavelet-HL_glszm_GrayLevelNonUniformityNormalized
wavelet-HL_glszm_GrayLevelVariance
wavelet-HL_glszm_HighGrayLevelZoneEmphasis
wavelet-HL_glszm_LargeAreaEmphasis
wavelet-HL_glszm_LargeAreaHighGrayLevelEmphasis
wavelet-HL_glszm_LargeAreaLowGrayLevelEmphasis
wavelet-HL_glszm_LowGrayLevelZoneEmphasis
wavelet-HL_glszm_SizeZoneNonUniformity
wavelet-HL_glszm_SizeZoneNonUniformityNormalized
wavelet-HL_glszm_SmallAreaEmphasis
wavelet-HL_glszm_SmallAreaHighGrayLevelEmphasis
wavelet-HL_glszm_SmallAreaLowGrayLevelEmphasis
wavelet-HL_glszm_ZoneEntropy
wavelet-HL_glszm_ZonePercentage

wavelet-HL_glszm_ZoneVariance
wavelet-HL_ngtdm_Busyness
wavelet-HL_ngtdm_Coarseness
wavelet-HL_ngtdm_Complexity
wavelet-HL_ngtdm_Contrast
wavelet-HL_ngtdm_Strength
wavelet-HH_firstorder_10Percentile
wavelet-HH_firstorder_90Percentile
wavelet-HH_firstorder_Energy
wavelet-HH_firstorder_Entropy
wavelet-HH_firstorder_InterquartileRange
wavelet-HH_firstorder_Kurtosis
wavelet-HH_firstorder_Maximum
wavelet-HH_firstorder_MeanAbsoluteDeviation
wavelet-HH_firstorder_Mean
wavelet-HH_firstorder_Median
wavelet-HH_firstorder_Minimum
wavelet-HH_firstorder_Range
wavelet-HH_firstorder_RobustMeanAbsoluteDeviation
wavelet-HH_firstorder_RootMeanSquared
wavelet-HH_firstorder_Skewness
wavelet-HH_firstorder_TotalEnergy
wavelet-HH_firstorder_Uniformity
wavelet-HH_firstorder_Variance
wavelet-HH_glcm_Autocorrelation
wavelet-HH_glcm_JointAverage
wavelet-HH_glcm_ClusterProminence
wavelet-HH_glcm_ClusterShade
wavelet-HH_glcm_ClusterTendency
wavelet-HH_glcm_Contrast
wavelet-HH_glcm_Correlation
wavelet-HH_glcm_DifferenceAverage
wavelet-HH_glcm_DifferenceEntropy
wavelet-HH_glcm_DifferenceVariance
wavelet-HH_glcm_JointEnergy
wavelet-HH_glcm_JointEntropy
wavelet-HH_glcm_Imc1
wavelet-HH_glcm_Imc2
wavelet-HH_glcm_Idm
wavelet-HH_glcm_Idmn

wavelet-HH_glcm_Id
wavelet-HH_glcm_Idn
wavelet-HH_glcm_InverseVariance
wavelet-HH_glcm_MaximumProbability
wavelet-HH_glcm_SumEntropy
wavelet-HH_glcm_SumSquares
wavelet-HH_gldm_DependenceEntropy
wavelet-HH_gldm_DependenceNonUniformity
wavelet-HH_gldm_DependenceNonUniformityNormalized
wavelet-HH_gldm_DependenceVariance
wavelet-HH_gldm_GrayLevelNonUniformity
wavelet-HH_gldm_GrayLevelVariance
wavelet-HH_gldm_HighGrayLevelEmphasis
wavelet-HH_gldm_LargeDependenceEmphasis
wavelet-HH_gldm_LargeDependenceHighGrayLevelEmphasis
wavelet-HH_gldm_LargeDependenceLowGrayLevelEmphasis
wavelet-HH_gldm_LowGrayLevelEmphasis
wavelet-HH_gldm_SmallDependenceEmphasis
wavelet-HH_gldm_SmallDependenceHighGrayLevelEmphasis
wavelet-HH_gldm_SmallDependenceLowGrayLevelEmphasis
wavelet-HH_glrlm_GrayLevelNonUniformity
wavelet-HH_glrlm_GrayLevelNonUniformityNormalized
wavelet-HH_glrlm_GrayLevelVariance
wavelet-HH_glrlm_HighGrayLevelRunEmphasis
wavelet-HH_glrlm_LongRunEmphasis
wavelet-HH_glrlm_LongRunHighGrayLevelEmphasis
wavelet-HH_glrlm_LongRunLowGrayLevelEmphasis
wavelet-HH_glrlm_LowGrayLevelRunEmphasis
wavelet-HH_glrlm_RunEntropy
wavelet-HH_glrlm_RunLengthNonUniformity
wavelet-HH_glrlm_RunLengthNonUniformityNormalized
wavelet-HH_glrlm_RunPercentage
wavelet-HH_glrlm_RunVariance
wavelet-HH_glrlm_ShortRunEmphasis
wavelet-HH_glrlm_ShortRunHighGrayLevelEmphasis
wavelet-HH_glrlm_ShortRunLowGrayLevelEmphasis
wavelet-HH_glszm_GrayLevelNonUniformity
wavelet-HH_glszm_GrayLevelNonUniformityNormalized
wavelet-HH_glszm_GrayLevelVariance
wavelet-HH_glszm_HighGrayLevelZoneEmphasis

wavelet-HH_glszm_LargeAreaEmphasis
wavelet-HH_glszm_LargeAreaHighGrayLevelEmphasis
wavelet-HH_glszm_LargeAreaLowGrayLevelEmphasis
wavelet-HH_glszm_LowGrayLevelZoneEmphasis
wavelet-HH_glszm_SizeZoneNonUniformity
wavelet-HH_glszm_SizeZoneNonUniformityNormalized
wavelet-HH_glszm_SmallAreaEmphasis
wavelet-HH_glszm_SmallAreaHighGrayLevelEmphasis
wavelet-HH_glszm_SmallAreaLowGrayLevelEmphasis
wavelet-HH_glszm_ZoneEntropy
wavelet-HH_glszm_ZonePercentage
wavelet-HH_glszm_ZoneVariance
wavelet-HH_ngtdm_Busyness
wavelet-HH_ngtdm_Coarseness
wavelet-HH_ngtdm_Complexity
wavelet-HH_ngtdm_Contrast
wavelet-HH_ngtdm_Strength
wavelet-LL_firstorder_10Percentile
wavelet-LL_firstorder_90Percentile
wavelet-LL_firstorder_Energy
wavelet-LL_firstorder_Entropy
wavelet-LL_firstorder_InterquartileRange
wavelet-LL_firstorder_Kurtosis
wavelet-LL_firstorder_Maximum
wavelet-LL_firstorder_MeanAbsoluteDeviation
wavelet-LL_firstorder_Mean
wavelet-LL_firstorder_Median
wavelet-LL_firstorder_Minimum
wavelet-LL_firstorder_Range
wavelet-LL_firstorder_RobustMeanAbsoluteDeviation
wavelet-LL_firstorder_RootMeanSquared
wavelet-LL_firstorder_Skewness
wavelet-LL_firstorder_TotalEnergy
wavelet-LL_firstorder_Uniformity
wavelet-LL_firstorder_Variance
wavelet-LL_glcm_Autocorrelation
wavelet-LL_glcm_JointAverage
wavelet-LL_glcm_ClusterProminence
wavelet-LL_glcm_ClusterShade
wavelet-LL_glcm_ClusterTendency

wavelet-LL_glcm_Contrast
wavelet-LL_glcm_Correlation
wavelet-LL_glcm_DifferenceAverage
wavelet-LL_glcm_DifferenceEntropy
wavelet-LL_glcm_DifferenceVariance
wavelet-LL_glcm_JointEnergy
wavelet-LL_glcm_JointEntropy
wavelet-LL_glcm_Imc1
wavelet-LL_glcm_Imc2
wavelet-LL_glcm_Idm
wavelet-LL_glcm_Idmn
wavelet-LL_glcm_Id
wavelet-LL_glcm_Idn
wavelet-LL_glcm_InverseVariance
wavelet-LL_glcm_MaximumProbability
wavelet-LL_glcm_SumEntropy
wavelet-LL_glcm_SumSquares
wavelet-LL_gldm_DependenceEntropy
wavelet-LL_gldm_DependenceNonUniformity
wavelet-LL_gldm_DependenceNonUniformityNormalized
wavelet-LL_gldm_DependenceVariance
wavelet-LL_gldm_GrayLevelNonUniformity
wavelet-LL_gldm_GrayLevelVariance
wavelet-LL_gldm_HighGrayLevelEmphasis
wavelet-LL_gldm_LargeDependenceEmphasis
wavelet-LL_gldm_LargeDependenceHighGrayLevelEmphasis
wavelet-LL_gldm_LargeDependenceLowGrayLevelEmphasis
wavelet-LL_gldm_LowGrayLevelEmphasis
wavelet-LL_gldm_SmallDependenceEmphasis
wavelet-LL_gldm_SmallDependenceHighGrayLevelEmphasis
wavelet-LL_gldm_SmallDependenceLowGrayLevelEmphasis
wavelet-LL_glrlm_GrayLevelNonUniformity
wavelet-LL_glrlm_GrayLevelNonUniformityNormalized
wavelet-LL_glrlm_GrayLevelVariance
wavelet-LL_glrlm_HighGrayLevelRunEmphasis
wavelet-LL_glrlm_LongRunEmphasis
wavelet-LL_glrlm_LongRunHighGrayLevelEmphasis
wavelet-LL_glrlm_LongRunLowGrayLevelEmphasis
wavelet-LL_glrlm_LowGrayLevelRunEmphasis
wavelet-LL_glrlm_RunEntropy

wavelet-LL_glrlm_RunLengthNonUniformity
wavelet-LL_glrlm_RunLengthNonUniformityNormalized
wavelet-LL_glrlm_RunPercentage
wavelet-LL_glrlm_RunVariance
wavelet-LL_glrlm_ShortRunEmphasis
wavelet-LL_glrlm_ShortRunHighGrayLevelEmphasis
wavelet-LL_glrlm_ShortRunLowGrayLevelEmphasis
wavelet-LL_glszm_GrayLevelNonUniformity
wavelet-LL_glszm_GrayLevelNonUniformityNormalized
wavelet-LL_glszm_GrayLevelVariance
wavelet-LL_glszm_HighGrayLevelZoneEmphasis
wavelet-LL_glszm_LargeAreaEmphasis
wavelet-LL_glszm_LargeAreaHighGrayLevelEmphasis
wavelet-LL_glszm_LargeAreaLowGrayLevelEmphasis
wavelet-LL_glszm_LowGrayLevelZoneEmphasis
wavelet-LL_glszm_SizeZoneNonUniformity
wavelet-LL_glszm_SizeZoneNonUniformityNormalized
wavelet-LL_glszm_SmallAreaEmphasis
wavelet-LL_glszm_SmallAreaHighGrayLevelEmphasis
wavelet-LL_glszm_SmallAreaLowGrayLevelEmphasis
wavelet-LL_glszm_ZoneEntropy
wavelet-LL_glszm_ZonePercentage
wavelet-LL_glszm_ZoneVariance
wavelet-LL_ngtdm_Busyness
wavelet-LL_ngtdm_Coarseness
wavelet-LL_ngtdm_Complexity
wavelet-LL_ngtdm_Contrast
wavelet-LL_ngtdm_Strength
